# Online team-based electrocardiogram training in Haiti: evidence from the field

**DOI:** 10.1186/s12909-022-03421-8

**Published:** 2022-05-11

**Authors:** Dawson Calixte, Norrisa Adrianna Haynes, Merly Robert, Cassandre Edmond, Lily D. Yan, Kate Raiti-Palazzolo, Evyrna Toussaint, Benito D. Isaac, Darius L. Fenelon, Gene F. Kwan

**Affiliations:** 1 Zanmi Lasante, Port Au Prince, Haiti; 2 University of Pennsylvania, Philadelphia, PA USA; 3 Weill Cornell Medicine, New York, USA; 4 Mount Sinai Hospital, New York, USA; 5 Section of Cardiovascular Medicine, Boston University School of Medicine, 72 East Concord St, Boston, MA D-808 USA; 6 Department of Global Health and Social Medicine, Harvard Medical School, Boston, MA USA; 7 Partners In Health, Boston, MA USA

**Keywords:** Haiti, ECG competition, Global health, Peer-to-peer learning

## Abstract

**Background:**

The electrocardiogram (ECG) is the most relied upon tool for cardiovascular diagnosis, especially in low-resource settings because of its low cost and straightforward usability. It is imperative that internal medicine (IM) and emergency medicine (EM) specialists are competent in ECG interpretation. Our study was designed to improve proficiency in ECG interpretation through a competition among IM and EM residents at a teaching hospital in rural central Haiti in which over 40% of all admissions are due to CVD.

**Methodology:**

The 33 participants included 17 EM residents and 16 IM residents from each residency year at the Hôpital Universitaire de Mirebalais (HUM). Residents were divided into 11 groups of 3 participants with a representative from each residency year and were given team-based online ECG quizzes to complete weekly. The format included 56 ECG cases distributed over 11 weeks, and each case had a pre-specified number of points based on abnormal findings and complexity. All ECG cases represented cardiovascular pathology in Haiti adapted from the Association of Program Directors in Internal Medicine evaluation list. The main intervention was sharing group performance and ECG solutions to all participants each week to promote competition and self-study without specific feedback or discussion by experts. To assess impact, pre- and post-intervention assessments measuring content knowledge and comfort for each participant were performed.

**Results:**

Overall group participation was heterogeneous with groups participating a median of 54.5% of the weeks (range 0–100%). 22 residents completed the pre- and post-test assessments. The mean pre- and post-intervention assessment knowledge scores improved from 27.3% to 41.7% (*p* = 0.004). 70% of participants improved their test scores. The proportion of participants who reported comfort with ECG interpretation increased from 57.6% to 66.7% (*p* = 0.015).

**Conclusion:**

This study demonstrates improvement in ECG interpretation through a team-based, asynchronous ECG competition approach. This method is easily scalable and could help to fill gaps in ECG learning. This approach can be delivered to other hospitals both in and outside Haiti. Further adaptations are needed to improve weekly group participation.

**Supplementary Information:**

The online version contains supplementary material available at 10.1186/s12909-022-03421-8.

## Background

The electrocardiogram (ECG) remains one of the most widely used diagnostic tools in cardiovascular medicine [1]. The ECG records the electrical activity of the heart and is frequently used to diagnose heart disease and electrolyte abnormalities. In low-resource settings, ECG is often the only or most efficient tool to identify life-threatening cardiac conditions enabling timely care. The burden of cardiovascular disease (CVD) is rising exponentially around the world but especially in low- and middle-income countries (LMICs) such as Haiti [2, 3]. Thus given the strikingly high burden of CVD and the diagnostic value of ECGs, the ability to correctly interpret ECGs is vitally important to the delivery of high quality healthcare [4].

As in most countries, ECG interpretation in Haiti is an important component of undergraduate and postgraduate medical training. Basic ECG interpretation is also incorporated into the curricula for trainees in internal medicine and emergency medicine training programs. The most commonly utilized methods for ECG instruction include didactic lectures and clinical teaching rounds [5, 6]. However, despite these methods, there is a lack of ECG interpretation proficiency among medical trainees [4, 7–10]. While in-person teaching remains the educational gold-standard, there is a dearth of cardiovascular subspecialists and educators in low-resource settings such as Haiti. The ability to provide high quality and consistent ECG teaching is a significant challenge.

The use of virtual learning for the delivery of medical education has become increasingly common especially during the COVID-19 era. Virtual education strategies allow for flexibility in the timing of delivery of educational content as well as the timing of active participation. In low-resource settings, it may also help address the lack of adequately trained educators. Studies suggest that using quizzes to facilitate ECG training via a web based model could be beneficial to trainees [11]. Additionally, Competition Based Learning has been shown to improve student motivation and performance in certain settings and contexts [12]. The use of a competitive, team-based, asynchronous, virtual approach to ECG interpretation education in Haiti has not been previously described.

Here we present a prospective cohort study evaluating the effectiveness of using a team-based, virtual ECG interpretation competition strategy to improve ECG interpretation skills among internal medicine (IM) and emergency medicine (EM) residents at Hôpital Universitaire de Mirebalais (HUM). HUM is one of the largest teaching hospitals in Haiti and is the only one to receive accreditation from the Accreditation Council for Graduate Medical Education International [13].

## Methods

### Setting and population

The Hôpital Universitaire de Mirebalais (HUM) was created in partnership between the Ministry of Public Health and Zanmi Lasante/Partners In Health. The hospital is located in Haiti’s rural Central Plateau and delivers acute care services to a broad catchment area with a population of about 3 million people. HUM offers high-quality residency training in emergency medicine, internal medicine, general surgery, plastic surgery, pediatrics, obstetrics and gynecology, and neurology.

The study was conducted over an 11 week period from November 11, 2019 to January 27, 2020 among IM and EM residents at HUM. Participation in the study was voluntary. In total, 33 residents including 16 IM residents and 17 EM residents were recruited for the study. Residents were divided into 11 groups of 3 participants. Groups were formed only within residency training programs. Each group consisted of one resident from each training year: postgraduate year (PGY) 1, 2, and 3. Further, we randomly assigned a unique identification number to each group.

This study was reviewed by the institutional review boards of Zanmi Lasante and Boston University Medical Campus and determined to qualify for an exempt determination. All participants reviewed a statement describing this educational research study as exempt.

### Study design

**Fig. 1:**
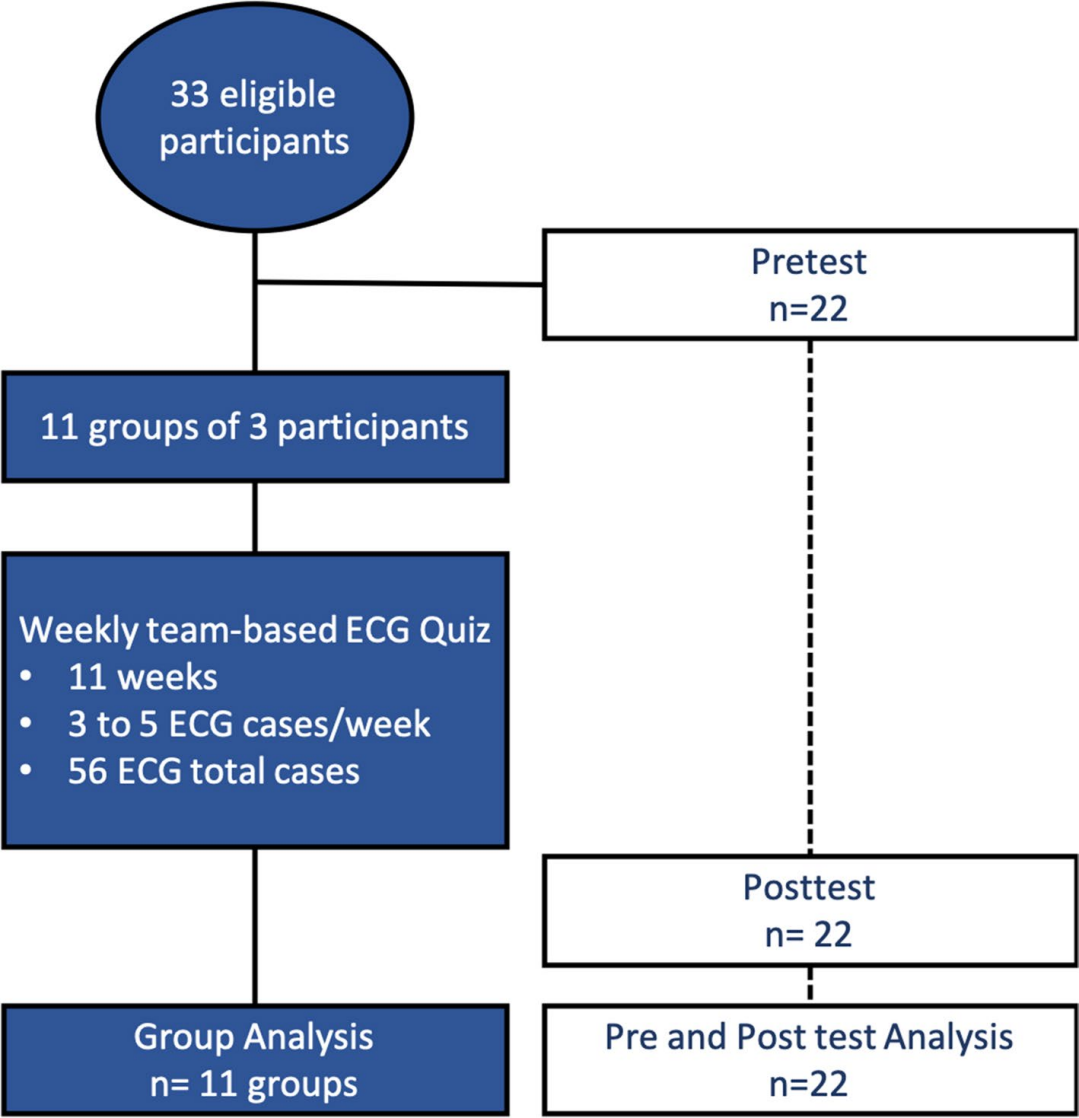
Study Design. Figure developed using Visme Software and is our own

We administered pretest and posttest evaluations to each participant to assess (1) the learning impact of the competition, and (2) comfort with independent ECG interpretation. We assessed ECG interpretation competency using 10 ECG cases with a maximum score of 35 points. The pretest and posttest were identical. We assessed comfort interpreting ECGs through a 5-point Likert scale: strongly agree to strongly disagree. Secondary outcomes included a change in self-reported comfort with ECG interpretation: “I am very comfortable interpreting ECGs”, with answer choices from Strongly Agree to Strongly Disagree (5-point Likert scale). The study design is presented in Fig. 1.

Ten ECG cases were administered in both pre-and-posttests; 56 ECG cases were distributed over 11 weeks.

After the pretest, we conducted the group-based competition intervention. Every week, each group received a web link to access an online folder containing PDF files of 3–5 ECG cases. Each ECG case included a clinical vignette with an image of an ECG, and diagnostic questions. All ECG cases and answers used for the study were selected from the textbook Podrid’s Real-World ECGs, Vol 1–6. Printed copies of the ECGs were also available. The selected ECGs cases were representative of common cardiovascular pathology seen both in the IM wards and the EM department at HUM adapted from the from the Association of Program Directors in Internal Medicine evaluation list. ECG topics included atrial fibrillation, atrial flutter, atrial paced rhythm, atrioventricular delay, mobitz type 1, mobitz 2, complete heart block, bundle branch blocks, ventricular premature complexes, ventricular tachycardia (VT), ventricular fibrillation, ischemia/infarction, and electrolyte abnormalities (Supplementary Material 1). Case difficulty was random with most of the last weeks' cases being hard. For future ECG training, one could recommend to start with easy cases and gradually move to more difficult cases. The clinical accuracy and appropriateness of the ECG cases and answers were independently reviewed by a cardiologist (GFK). Trainees did not receive any specific materials to guide their analysis of the weekly ECG. Trainees could use any available resource including textbooks and web-based content.

For the first 2 weeks, each group received 5 ECG cases to test their competency in ECG reading and correctly diagnosing the cases used in the study. Based on participant feedback that the weekly quizes were too long, we decreased the number of ECG cases to 3 ECG cases per week for the remainder of the study. In total, we distributed 56 ECG cases. For each case, participants had to select the relevant diagnoses on a standardized answer sheet adapted from the American Board of Internal Medicine Cardiovascular Disease Certification Exam (Supplementary Material 2). Each group received a web link to record their answers in an electronic REDCap database.

We classified the ECG cases on a scale from easy to hard based on the learning objectives and the number of abnormalities. ECGs with more abnormalities had a higher maximum score. The total weekly maximum scores varied from 7 to 20 points. Each ECG abnormality was scored as either correct or incorrect. We did not penalize participants for incorrect responses and there was no limit to the number of selected answers for each ECG case. The scores were summed together for each group.

During the first week of the study, we shared the quiz scores of each group to all the teams via email. After feedback from participants revealed that the public posting of each group's single score was disconcerting to participants, we modified the protocol. In the revised protocol, only the maximum and the minimum scores blinded to group identity were sent to each group via email to increase motivation and promote engagement. Thus, each group could assess their performance relative to their peers. After each weekly quiz, we distributed the correct responses and detailed descriptions with annotated ECGs to the trainees. Trainees could review these solutions independently.

### Statistical analysis

The primary outcome was a change in individual scores on the pre- and post-test knowledge assessments. For comparison of pre- and post-intervention mean objective assessment scores, a paired 2-sample t-test analysis was performed on the percent correct. Likert scale answer choices were used to assess secondary outcomes. For comparison of Likert scale scores, chi square analyses were performed. Fidelity was determined by the percent of weekly quizzes completed by the groups to determine high vs. low participation. Scores across weekly quizzes were also compared. In all analyses, differences were considered statistically significant when the *p*-value was less than 0.05. Statistical analysis was performed using Stata software 12.1.

## Results

### Pre- and post-test

**Fig. 2:**
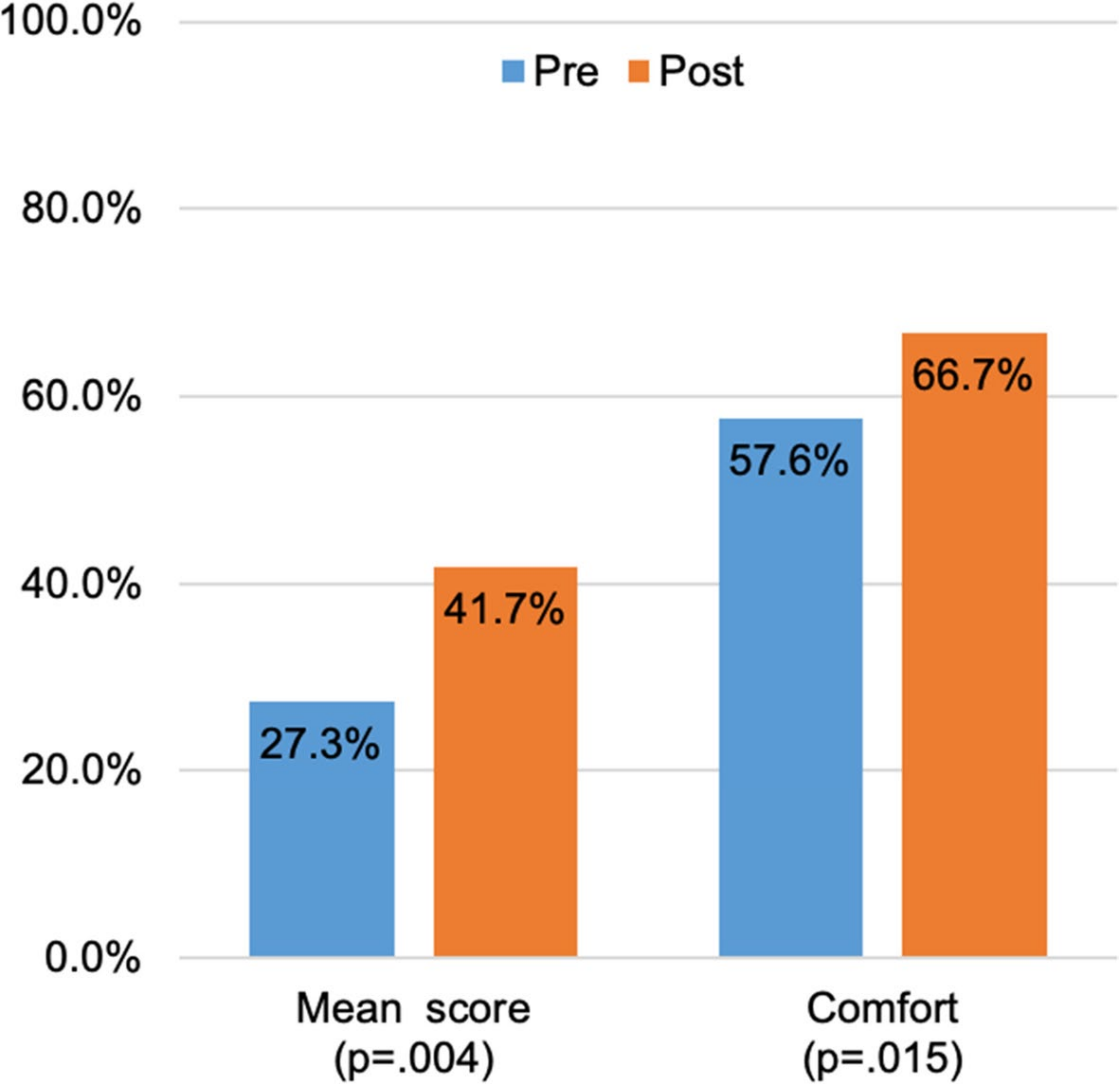
Mean test scores and comfort level with ECG interpretation improve pre- vs. post-intervention. Figure developed using Microsoft Excel and data are our own

A total of 33 residents participated in the study in 11 different groups, 16 internal medicine (IM) and 17 emergency medicine (EM) residents. Baseline characteristics of the participants are reported in Table 1. 22 residents completed both the pretest and posttest, both of which were out of 35 total points. The mean pretest score was 27.3% [standard deviation 15.6%], while the mean posttest score was 41.7% [sd 24.9%], for a statistically significant increase of 14.4 percentage points [*p* = 0.004] (Fig. 2).

**Table 1:** Participant characteristics and test performance

Residency	Emergency Medicine			Internal Medicine			All		
Resident year	**n**	**Pretest**	**Posttest**	**n**	**Pretest**	**Posttest**	**N**	**Pretest**	**Posttest**
PGY1	6	21.4%	38.6%	5	25.7%	30.3%	11	23.4%	34.8%
PGY2	2	7.1%	24.3%	1	45.7%	60.0%	3	20.0%	36.2%
PGY3	4	30.0%	60.0%	4	40.7%	46.4%	8	35.4%	53.2%
Total	12	21.9%	43.3%	10	33.7%	39.7%	22	27.3%	41.7%

Data for participants with both pre- and post-test results shown. Mean test results shown

### Weekly quizzes

The participation of groups across weeks ranged from no participation (0 weeks) among 2 groups to full participation (11 weeks) among 3 groups. 5 groups had low participation (= < 5 weeks), while 5 groups had high participation (> = 6 weeks). Weekly scores varied greatly from week-to-week, and between groups (Supplementary Material 3).

**Fig. 3:**
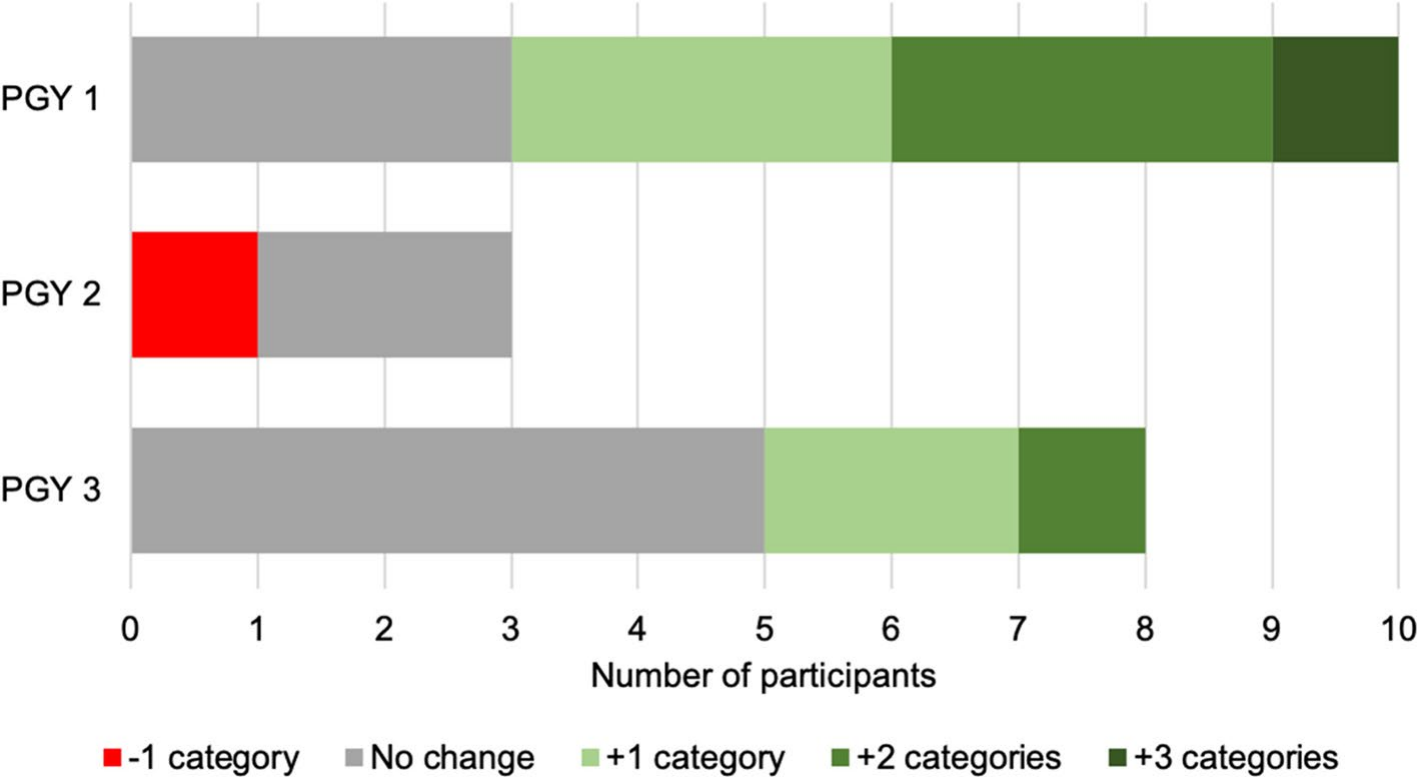
Change in comfort with interpreting ECGs by residency year. Comfort was assessed using a 5-point Likert scale. Positive values indicate improvement in comfort while negative values indicate reduction in comfort. The difference in comfort level between training years was not statistically significant (*p* = 0.473). PGY = post graduate year. Figure developed using Microsoft Excel and data are our own

Resident comfort increased over the course of the study. Pre-intervention, 57.6% (19/33) of respondents reported feeling comfortable (Strongly Agree, Agree) interpreting ECGs compared to 66.7% (22/33) post-intervention (*p* = 0.015). There was no significant difference in comfort with ECG interpretation between IM and EM residents post-intervention. Additionally, there was no significant difference in comfort with ECG interpretation post-intervention based on training year. Despite not reaching statistical significance, first year residents demonstrated a numerically larger improvement in ECG interpretation comfort compared to 2nd and 3rd year residents (Fig. 3). When looking at participation, 6 out of 11 groups were higher fidelity groups (defined as groups that completed greater than 50% of the weekly quizzes) and they reported feeling more comfortable with ECG interpretation compared to the 5 lower fidelity groups, although this difference did not reach statistical significance.

## Discussion

This virtual asynchronous ECG competition strategy improved residents’ ability to accurately interpret ECGs. Seventy percent of participants significantly improved their ECG interpretation scores post-intervention. In addition, the study also demonstrated a significant improvement in residents’ comfort interpreting ECGs.

The findings of this study are important given the rising burden of CVD in Haiti, and the diagnostic value of ECGs. Additionally, the findings highlight the value of innovative and adaptive teaching methodologies. Studies have demonstrated that effective teaching and improvement in trainee performance is heavily dependent on using different and versatile learning styles [11, 14–16]. Our team-based ECG competition strategy provided flexibility for learners to access educational materials and learn at their convenience based on clinical and personal responsibilities. In this regard, it served as a valuable adjunct to in-person learning. Studies have shown that web-based learning strategies, such as the intervention used in this study, are effective at overcoming barriers imposed by distance, time constraints, and resource limitations, and also enable implementation of novel instructional methods [17].

We used a very detailed scoring system for ECG analysis – which the participants were not accustomed to using. A trainee may feel comfortable assessing for major abnormalities, but not minor ones. Thus, trainees reported relatively high comfort with ECG interpretation at baseline despite relatively low pre-test scores. In our scoring system, trainees did not earn points for missing either major or minor abnormalities.

Importantly, an element of competition was used as a motivating factor in this intervention. Studies have shown that the use of competition and quizzes in teaching interventions can improve motivation and performance in certain situations [12, 16]. Competition, however, may also increase stress and performance anxiety among some students which may lead to reduced effort and knowledge retention [12, 18]. In this study, while the team-work component of the intervention was well received and facilitated peer-to-peer learning, the competition element characterized by the public display of group scores was not. Through informal feedback, participants expressed concern about the public display of group scores stating that this form of competition invoked stress and embarrassment for some of the participants. Thus, we adjusted the competition component of the study such that the scores for each group were not displayed but instead, the highest and lowest scores for each quiz were displayed without reference to any particular group. This modification was important because it continued to motivate participants to improve their scores but eliminated the possibility of identification and by doing so eliminated associated stress and embarrassment. With this modification, participants had significant improvements in their objective and comfort assessments scores demonstrating the efficacy of this web-based ECG competition intervention.

Although the improvement in ECG interpretation for the study cohort en masse was significant, subgroup analyses comparing training programs (IM vs. ED) and training years (PGY1 vs. PGY2 vs. PGY3), did not demonstrate a statistically significant difference between the groups. One explanation could be the small sample size and lack of power to detect a difference as well as trainee dropout. Additionally, six groups met high fidelity criteria defined as participation >  = 6 weeks. Some residents may have been dropout and some groups may have had low fidelity due to lack of familiarity with REDcap since it is a new system that the residents may find difficult to navigate. Of the 3 PGY2 residents who completed the survey, one participant reported less comfort after the intervention while the others had no change. This worsening of comfort may be due to low participation among this group of residents with very high clinical workload. The low fidelity and dropout may also be a function of a real world application of a voluntary medical education intervention among physicians-in-training with demanding clinical schedules.

This is the first study to assess the efficacy of a web-based ECG competition strategy in improving ECG interpretation proficiency in Haiti. This study is a successful proof of concept that demonstrates that this web-based ECG competition teaching strategy is feasible and can be effective. It is also easily scalable and can be incorporated into the training of health professions at additional sites in Haïti and in other LICs.

### Limitations

Limitations of this study include a small sample size, dropout among residents which may have been due, in part, to a lack of familiarity with REDcap. Further, lack of a non-intervention group for comparison limits our ability to detect expected improvement in skills over time among trainees. Additionally, the improvement in scores may, in part, be due to a training effect possibly caused by identical ECGs in pretest and posttest. Our ability to assess the contribution of the team-based nature of the intervention is limited. While we intended for the team participants to work collaboratively each week, we do not know how the teams functioned. Each residency does have regular teaching sessions where trainees can meet face-to-face. However, there could have been one dominant participant from a group, or answers submitted in a non-collaborative manner. Further trainees on off-site rotations may not be able to meet face-to-face.

## Conclusion

This study demonstrates the feasibility and efficacy of a team-based, asynchronous competition approach to ECG education. Given the success of this pilot, medical educators should consider this strategy in their educational armamentarium. Further studies are needed to improve participation, assess generalizability, and knowledge retention.

## Supplementary Information

**Additional file 1. **ECG topics with their level of difficulty.

**Additional file 2. **Quiz Answer sheet.

**Additional file 3. **Weekly quiz score by group.

**Additional file 4. **

## Data Availability

The datasets used and/or analysed during the current study are included in this published article as a supplement (Additional file 4).
