# Supplementary material for: Online team-based electrocardiogram training in Haiti: evidence from the field
Source: BMC Med Educ. 2022 May 11;22:360. doi: 10.1186/s12909-022-03421-8 (PMC9094130; doi:10.1186/s12909-022-03421-8)
Supplement: Supplementary file 1 — Additional file 1. ECG topics with their level of difficulty. [file 12909_2022_3421_MOESM1_ESM.docx]

Supplementary Material 1: ECG topics with their level of difficulty
ECG topics, level of difficulty, abnormalities and week number are presented in this table

| **ECG TOPICS** | **LEVEL OF DIFFICULTY** | **ABNORMALITIES** | **WEEK** |
| --- | --- | --- | --- |
| Normal ECG | Easy | Normal | 1 |
| Atrial Flutter | Hard | Abnormal | 1 |
| Ventricular premature complexes | Easy | Abnormal | 1 |
| Ventricular Tachycardia | Easy | Abnormal | 1 |
| ST and/or T waves ischemia | Easy | Abnormal | 1 |
| Atrial Fibrillation | Easy | Abnormal | 2 |
| Sinus bradycardia | Hard | Abnormal | 2 |
| Mobitz type 1 second degree AV block | Hard | Abnormal | 2 |
| Atrial paced rhythm | Hard | Abnormal | 2 |
| Ventricular fibrillation | Easy | Abnormal | 2 |
| First degree heart block | Easy | Abnormal | 3 |
| Left atrial enlargement, abnormality or conduction defect | Hard | Abnormal | 3 |
| Atrial premature complexes, conducted | Easy | Abnormal | 3 |
| Artifact | Hard | Abnormal | 3 |
| Right axis deviation | Hard | Abnormal | 3 |
| AV dissociation | Hard | Abnormal | 4 |
| Right bundle branch block | Easy | Abnormal | 4 |
| Mobitz type 2 second degree AV block | Hard | Abnormal | 4 |
| Ectopic atrial tachycardia, unifocal | Hard | Abnormal | 4 |
| Leads misplaced | Hard | Abnormal | 4 |
| Inferior MI (acute or recent) | Easy | Abnormal | 5 |
| Hyperkalemia | Hard | Abnormal | 5 |
| Left bundle branch block | Easy | Abnormal | 5 |
| Atrial premature complexes, non-conducted | Hard | Abnormal | 5 |
| Ectopic atrial tachycardia, multifocal | Hard | Abnormal | 5 |
| AV block complete (third degree) | Hard | Abnormal | 6 |
| Ventricular tachycardia, polymorphic (including Torsades de pointes) | Easy | Abnormal | 6 |
| Supraventricular tachycardia (paroxysmal) | Easy | Abnormal | 6 |
| Lateral MI (old) | Hard | Abnormal | 6 |
| Atrial sense ventricular paced | Hard | Abnormal | 6 |
| Sinus arrest or pause | Easy | Abnormal | 7 |
| Hypercalcemia | Hard | Abnormal | 7 |
| AV block or conduction ratio, 2:1 | Hard | Abnormal | 7 |
| Indeterminate axis | Hard | Abnormal | 7 |
| Low voltage (<0.5 Mv total QRS amplitude in each ex | Easy | Abnormal | 7 |
| Anterior MI (acute or recent) | Easy | Abnormal | 8 |
| Ventricular paced rhythm | Hard | Abnormal | 8 |
| Right axis deviation | Hard | Abnormal | 8 |
| Left anterior fascicular block | Hard | Abnormal | 8 |
| Anterior MI (old) | Easy | Abnormal | 8 |
| Electrical alternant | Hard | Abnormal | 9 |
| Chronic pulmonary disease pattern | Hard | Abnormal | 9 |
| Sinus tachycardia | Hard | Abnormal | 9 |
| Aberrant conduction of supraventricular beats | Hard | Abnormal | 9 |
| Sinus rhythm | Hard | Abnormal | 9 |
| Hypokalemia | Hard | Abnormal | 10 |
| QT interval prolonged | Hard | Abnormal | 10 |
| Ventricular pre-excitation (Wolff-Parkinson White) | Hard | Abnormal | 10 |
| Sinus arrest or pause | Hard | Abnormal | 10 |
| Hypocalcemia | Hard | Abnormal | 10 |
| Juvenile T waves (normal variant) | Hard | Abnormal | 10 |
| ST suggest ischemia | Hard | Abnormal | 11 |
| Acute pericarditis | Hard | Abnormal | 11 |
| AV sequential pacing | Hard | Abnormal | 11 |
| Left axis deviation (-30 to -90 degrees) | Hard | Abnormal | 11 |
| Septal MI (old) | Easy | Abnormal | 11 |
