# Supplementary material for: Online team-based electrocardiogram training in Haiti: evidence from the field
Source: BMC Med Educ. 2022 May 11;22:360. doi: 10.1186/s12909-022-03421-8 (PMC9094130; doi:10.1186/s12909-022-03421-8)
Supplement: Supplementary file 2 — Additional file 2. Quiz Answer sheet. [file 12909_2022_3421_MOESM2_ESM.docx]

Supplementary Material 2: Quiz Answer sheet
The participants were asked to record their responses on the answer sheet

\

Name:

ECG Number:

**ECG Coding Sheet**

**RATE AND RHYTHM**

- Normal ECG
- Sinus rhythm
- Sinus bradycardia (<60)
- Sinus tachycardia (>100)
- Sinus pause or arrest
- Atrial premature complexes, conducted
- Atrial premature complexes, nonconducted
- Atrial tachycardia
- Atrial tachycardia, multifocal
- Supraventricular tachycardia, paroxysmal
- Atrial flutter
- Atrial fibrillation
- Junctional escape complexes
- Ventricular premature complexes
- Ventricular tachycardia (>3 consecutive complexes)
- Ventricular tachycardia, polymorphic (including Torsades)
- Ventricular escape complexes or rhythm
- Ventricular fibrillation

**CONDUCTION**

- AV delay (1st degree AVB)
- AV block, 2nd degree, Mobitz type I (Wenckebach)
- AV block, 2nd degree, Mobitz type II
- AV block, 2:1
- AV block, 3rd degree
- Ventricular pre-excitation, Wolff Parkinson White pattern
- AV dissociation
- Right bundle branch block, intermittent or fixed
- Left bundle branch block, intermittent or fixed
- Left anterior fascicular block
- Left posterior fascicular block
- Aberrant conduction of supraventricular beats
- Nonspecific intraventricular conduction disturbance

**INFARCTION, ISCHEMIA, AND ST SEGMENT ABNORMALITIES**

- Anterior or anteroseptal MI (age recent or acute)
- Anterior or anteroseptal MI (age indeterminate or old)
- Lateral MI (age recent or acute)
- Lateral MI (age indeterminate or old)
- Inferior MI (age recent or acute)
- Inferior MI (age indeterminate or old)
- Normal variant, early repolarization
- Normal variant, juvenile T waves
- Nonspecific ST and/or T wave abnormalities
- ST and/or T wave abnormalities suggesting myocardial ischemia
- ST and/or T wave abnormalities suggesting myocardial injury
- Prolonged QT interval

**AXIS, VOLTAGE, AND HYPERTROPHY**

- Right atrial abnormality/enlargement
- Left atrial abnormality/enlargement
- Left axis deviation (> -30 degrees)
- Right axis deviation (> +100 degrees)
- Electrical alternans
- Low voltage
- Right ventricular hypertrophy
- Left ventricular hypertrophy
