# Supplementary material for: Online team-based electrocardiogram training in Haiti: evidence from the field
Source: BMC Med Educ. 2022 May 11;22:360. doi: 10.1186/s12909-022-03421-8 (PMC9094130; doi:10.1186/s12909-022-03421-8)

Supplementary Material 3: Weekly quiz score by group.
Maximum score in a given week is 100%. Figure developed using STATA and data are our own.


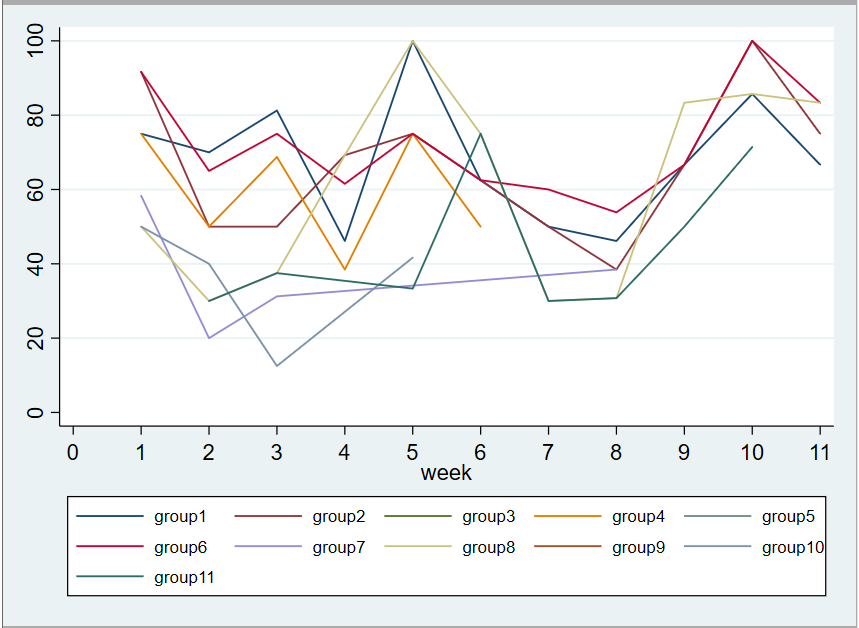

Supplement: Supplementary file 3 — Additional file 3. Weekly quiz score by group. [file 12909_2022_3421_MOESM3_ESM.docx]
